# Correlation Between the Oral Microbiota and Sports Practice: A Systematic Review

**DOI:** 10.7759/cureus.78168

**Published:** 2025-01-29

**Authors:** Margaux Dubois, Bérangère Delcourt, Morgane Ortis, Valérie Bougault, Alain Doglio, Marie-France Bertrand

**Affiliations:** 1 Laboratory of Oral Microbiology, Immunotherapy, and Health, University Côte d'Azur, Nice, FRA; 2 Department of Odontology, University Côte d'Azur, Nice, FRA; 3 Institute of Oral and Dental Medicine, Centre Hospitalier Universitaire de Nice (CHU de Nice), Nice, FRA; 4 Laboratory of Human Motricity, Expertise, Sport, and Health (LAMHESS), University Côte d'Azur, Nice, FRA; 5 Institute of Cell and Gene Therapy, Centre Hospitalier Universitaire de Nice (CHU de Nice), Nice, FRA

**Keywords:** oral health, oral health in athletes, oral microbiota, sports, sports performance

## Abstract

The impact of sports on oral health has been the subject of extensive investigation, with the majority of studies indicating a deterioration in oral health. However, the composition of the oral microbiota in athletes and its impact remains unclear. The objective of this review is to investigate the potential correlation between athletic activity and alterations in the oral microbiota. A comprehensive electronic search was conducted up to November 2024 across three different databases (PubMed, the Cochrane Library, and Scopus) with the objective of identifying studies that evaluate the association between oral microbiota and physical activity. Two independent blinding review authors were involved in study selection, data extraction, and bias assessment using the National Institutes of Health’s (NIH) study quality assessment tools. A total of 147 records were screened, and five eligible studies were included. Recent studies have demonstrated that individuals who engage in regular physical activity exhibit distinctive oral microbial composition in comparison to those with sedentary lifestyles or low levels of physical activity. Three studies have demonstrated that the athlete's oral microbiota is modified, with an increase in the genera *Rothia, Stenotrophomonas*, and *Veillonella*, and a decrease in the genus *Gemella*. The *Streptococcus* genus is often modified in athletes according to four studies. This review provided an analysis of the scientific evidence indicating that the oral microbiota of athletes is modified. But to date, there is no scientific evidence to clearly determine the impact of sports on these variations. More homogeneous studies with the limitation of bias are needed to better understand the link between sports and oral microbiota.

## Introduction and background

In a context where the pursuit of athletic performance continues to intensify, the bi-directional relationship between oral health and sports practice is receiving increasing attention, insofar as oral health is closely connected to the body’s general health [1]. Early research on the oral health of elite athletes dates back to the late 1960s [2], with a significant acceleration following the 2004 Athens Olympic Games. Notably, a high prevalence of oral health issues was documented at the dental clinics of both the Beijing 2008 and London 2012 Summer Olympic and Paralympic Games [3]. In 2015, a systematic review further confirmed the poor oral health of high-level athletes, revealing a frequent incidence of different oral diseases such as carious lesions, dental erosions, and irreversible periodontal diseases [4]. In accordance with these findings, Needleman et al. demonstrated an association between elite athletic status and poor oral health, comparable to the prevalence observed in socioeconomically disadvantaged non-athlete populations [5]. In particular, these authors highlighted the critical need for targeted interventions to address the specific oral health challenges faced by athletes, such as immune abnormalities associated with intense training, elevated stress levels in competitive environments, dietary practices involving supplements, the use of mouthguards, and insufficient oral hygiene [5, 6].

These factors are likely to have a disruptive effect on the complex oral ecosystem, more specifically regarding the oral microbiome, a highly complex community of microorganisms that colonize the various ecological niches within the oral cavity, including the teeth, gums, tongue, and cheeks [7]. The oral microbiota is highly diverse, with over 700 species of bacteria, but also viruses, fungi, and archaea, which interact closely with each other and with the host environment [8]. It is estimated to contain nearly 6x10^9^ microorganisms, making it the second most complex microbial ecosystem in the human body after the intestinal microbiota [9]. Maintaining a healthy oral microbiota is crucial for preventing oral diseases and beyond to ensure good general health. Disruption in oral microbiota homeostasis, a condition known as dysbiosis [10], is closely associated with a wide range of oral pathologies, including periodontitis, dental caries, and systemic pathologies such as diabetes, cardiovascular diseases, and respiratory infections [11]. Such disruptions may lead to an increased susceptibility to dental and periodontal diseases, further impacting overall well-being and athletic performance [1,12,13]. Better understanding the oral microbiota, particularly the dynamics of the oral biofilm and the role of various microbial species, is thus of critical importance for developing effective strategies to prevent oral diseases and enhance oral health [14].

While regular physical activity is generally associated with numerous health benefits, research suggests that competitive athletes often experience compromised oral health. However, the impact of sport on the oral microbiome remains a relatively underexplored area. This literature review aims to analyze the available studies on this subject and explore the potential relationship between sport and alterations in the oral microbiota.

## Review

Materials and methods

Identification of the Research Question

This systematic review was conducted in accordance with the Preferred Reporting Items for Systematic Reviews and Meta-Analyses (PRISMA) guidelines [15]. The study was registered in the International Prospective Register of Systematic Reviews (PROSPERO) under the registration number CRD42024550542.

The review adhered to the Population, Exposure, Comparison, and Outcomes (PECO) criteria which were as follows: (1) Population: adults; (2) Exposure: athletic participation; (3) Comparison: control group, non-athletes (4) Outcomes-analyzing the variation of the oral microbiota. The formulated PECO research question was “In adults (P), what is the effect of athletic participation (E) compared with non-athletes (C) on the oral microbiota (O)? If so, what is the nature of this change?”.

Eligibility Criteria

Prior to the screening process, a set of eligibility criteria was established. The inclusion criteria were as follows: (i) articles with an available abstract, (ii) articles published in English or French, and (iii) studies specifically investigating the composition of the oral microbiota and describing the relationship with sports practice. For the purposes of this study, physical activity was defined as a structured exercise involving regular training within an organized framework.

The exclusion criteria were: (i) studies focusing on populations with underlying pathologies, (ii) studies in which the analysis of the oral microbiota was insufficiently defined or inadequately described, and (iii) in vitro studies, systematic reviews, narrative reviews, and author opinion reviews.

Information Source and Search Strategy

An electronic search was carried out in November 2024 across three different databases: PubMed, the Cochrane Library, and Scopus, to identify eligible published studies. The following keywords used were “Sports (MeSH)”, “Athletic”, “Athletics”, “Athletic Performance (MeSH)”, “Microbiota (MeSH)”, “Oral Microbiota”, “Microbiome”, “Human Microbiome”, “Oral”, “Oral Health (MeSH)” and “Mouth (MeSH)”. These keywords were combined using Boolean operators (Table 1).

**Table 1:** Search strategy for the systematic review MeSH: Medical Subject Headings

Number	Search strategy
1	Sports (MeSH)
2	Athletic
3	Athletics
4	Athletic Performance (MeSH)
5	Microbiota (MeSH)
6	Oral Microbiota
7	Microbiome
8	Human Microbiome
9	Oral
10	Oral Health (MeSH)
11	Mouth (MeSH)
12	1 OR 2 OR 3 OR 4
13	5 OR 6 OR 7 OR 8
14	9 OR 10 OR 11
15	12 AND 13 AND 14

The search strategy employed was as follows: ((sports) OR (Athletics) OR (Athletic) OR (Athletic Performance)) AND ((Microbiota) OR (Oral Microbiota) OR (Microbiome) OR (Human Microbiome)) AND ((Oral) OR (Oral Health) OR (Mouth)). Only articles written in English or French were included, with no restrictions on the year of publication. An additional manual search was performed in the bibliography of each included article to identify potential articles not found by the electronic search.

Selection Process

Two authors (MD and BD) independently selected the articles and extracted the relevant data. First, duplicates were manually removed. Subsequently, a pre-selection of potentially relevant studies was conducted based on the screening of titles and abstracts. The final selection of articles for inclusion was determined by reviewing the full texts, in line with the PICO framework and the established inclusion and exclusion criteria. In cases of disagreement regarding article selection, a third author (MB) was consulted, and consensus was achieved through discussion among the authors.

Data Collection Process

The two authors (MD and BD) independently reviewed the eligible papers and extracted the following information: author, year and journal of publication, study design, inclusion and exclusion criteria, protocol description, and summary of results. Given the number of articles included and the heterogeneity of the protocols, methodological analyses, and results, a meta-analysis was not performed.

Risk of Bias

The risk of bias was assessed independently by two reviewers using the National Institutes of Health’s (NIH) study quality assessment tools for observational cohort and cross-sectional studies, as well as for case-control studies [16]. In cases of discrepancies between the reviewer’s assessments, a consensus was reached through discussion and reassessment. If necessary, the opinion of a third member of the review team was sought to resolve any remaining disagreements.

Results

**Figure 1:**
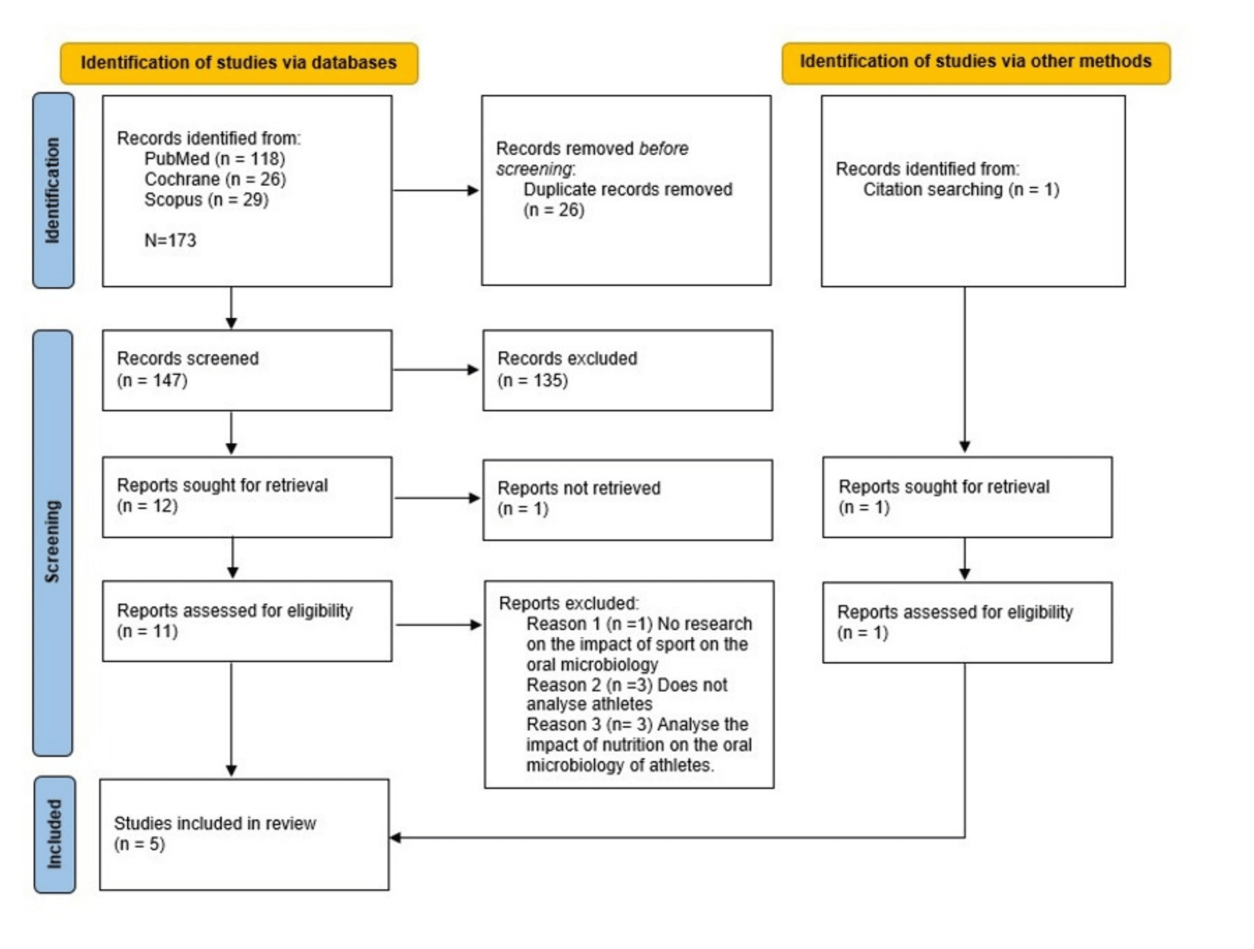
A PRISMA flowchart diagram describing the selection process PRISMA: Preferred Reporting Items for Systematic Reviews and Meta-Analyses; Cochrane: the Cochrane Library

From the electronic search across the three databases, a total of 173 records were identified. Following manual screening, 26 duplicate records were identified and removed. After reviewing the titles, 135 records were excluded as they did not meet the eligibility criteria. The full-text assessment was conducted on the remaining 12 records, resulting in the exclusion of eight articles for the following reasons: inability to retrieve the full text (n=1); lack of analysis on the oral microbiota (n=1); sample not including athletes or participants with training experience (n=3); and a focus on the impact of nutrition on athlete microbiology (n=3). Additionally, one study (n=1) was identified through citation searching of relevant articles. In total, five studies were included in the final analysis (Figure 1). These consisted of one cross-sectional study [17] and four case-control studies [18-21].

Study Characteristics

The general characteristics of the selected studies are summarized in Table 2.

**Table 2:** Characteristics of the selected studies NCAA: National Collegiate Athletic Association; PRG: professional rugby players; CG: control group; CFU/ml: colony-forming units per milliliter; PCR: polymerase chain reaction; ATCC: American Type Culture Collection

Authors and years	Type of study	Characteristics of participants	Method of analysis	Main results
Lamb et al., 2016 [18]	Case-control study	Sports group: 10 female students playing hockey during the sports season (active); Non-athlete group: nine students who played football during the off-season (inactive) at the time of the study. Characteristics: Students aged between 18 and 20 years in NCAA Division III at Chatham University, USA.	Bacterial growth on different agars was as follows: Blood agar showed non-specific growth; Mitis salivarius agar showed the growth of *Streptococcus salivarius* and *Streptococcus mitis*; *Staphylococcus *110 agar showed the growth of *Staphylococci*. The results were expressed in CFU/ml.	Blood agar: CFU/ml higher in athletes in season than out of season (p=0.045). Mitis salivarius agar: A statistically significant decrease in the number of bacteria in the in-season group (p=0.0006). *Staphylococcus *110 agar: No significant difference in the number of CFU/ml between the two groups (p = 0.506).
Minty et al., 2018 [19]	Case-control study	Sports group (PRG): 24 professional rugby players from the Union Sportive Colomiers team, France; Non-athlete group (CG): 22 sedentary people from south-west France	Bacterial growth on sheep blood agar; 16s rDNA sequencing to define the salivary bacterial composition	Sheep's blood agar: The number of total CFUs decreased in elite rugby players. Sequencing: Increase in *Streptococcus *species (p=0.005) particularly those involved in caries in PRGs (*Streptococcus mutan*s*, Streptococcus thermophilus, Streptococcus sobrinus, *and *Streptococcus gordonii *and the *Rothia *genus (p<0.0001)). The oral microbiota of the GC was dominated by the genera *Proteobacteria*, *Bacteroidetes, Neisseria, Porphyromonas, Haemophilu*s, and *Fusobacterium *compared with the PRG.
Basak et al., 2015 [17]	Cross-sectional study	Cohort: 20 Turkish professional swimmers; 14 men and six women aged between 15 and 30 years. Samples taken before and after training	16s rDNA sequencing to define the salivary bacterial composition	The number of *Bacteroides *(p = 0.007) and *Firmicutes *(p = 0.014) phyla was reduced after training. The *Bacilli *(p = 0.048) and *Clostridia *(p = 0.006) classes were reduced after training. The orders *Clostridiales *(p= 0.004), *Entomoplasmatales *(p=0.009), and *Bacillales *(p=0.006) were reduced after training. The bacterial family *Lachnospiraceae *(p=0.001) was reduced after training. The genus *Stenotrophomonas *(p=0.013) increased after training. The genera *Streptococcus *(p=0.232), *Pseudomonas *(p=0.526), *Serratia* (p=1.000), *Chryseobacterium *(p=0.372), and *Rothia *(p=0.052) were higher than the other genera detected but did not vary significantly after training.
Kalabiska et al., 2023 [20]	Case-control	Sports group: 29 water polo players; Non-athlete group: 16 sedentary people (eight men and eight women); Characteristics: participants aged between six and 20 years. Study carried out in Hungary.	16s rDNA sequencing to define the salivary bacterial composition	Men had a higher abundance of the genera *Atopobium *and* Prevotella_7* (p < 0.05). Men had a lower abundance of the genera *Fusobacterium, Gemella,* and *Streptococcus *(p < 0.05). Compared with the control group, water polo players had a significantly higher relative abundance of the genus *Veillonella *(p < 0.05, 18.3% vs. 14.6%) and a significantly lower relative abundance of the genus *Gemella *(p < 0.05, 3.8% vs. 4.9%).
D’ercole et al., 2015 [21]	Case-control	Sports group: 54 competitive swimmers; Non-athlete group: 69 non-competitive swimmers; Characteristics: a cohort of adolescents, two samples taken before and after training was carried out in Italy.	PCR of three species of oral streptococci: *Streptococcus mutans *ATCC31383, *Streptococcus sobrinus *ATCC27607, and *Streptococcus sanguinis *49297	Of the group, 18.64% of competitive swimmers and 32.2% of control swimmers had *Streptococcus mutans *in their saliva (p > 0.05). *Streptococcus sobrinus* was present in 22.03% of competitive swimmers and 91.6% of non-competitive swimmers (p < 0.05). On the other hand, *Streptococcus sanguinis *was only detected in the saliva sample of competitive swimmers. swimmers. There was no difference between the pre- and post-training samples.

The included studies were published between 2015 and 2023, indicating a relatively recent body of research. These studies were conducted in various countries, including the United States [18], France [19], Türkiye [17], Hungary [20], and Italy [21].

Two studies focused on team sports, specifically rugby, hockey, and football, while three studies examined water sports, such as swimming and water polo. The populations studied varied across the research. In the case-control studies, two studies compared groups of athletes to sedentary individuals, one study analyzed differences between active and inactive athletes, and one study compared competitive athletes with non-competitive athletes. The cross-sectional study assessed changes within a single group of athletes before and after training.

A total of 332 participants aged between six and 32 years were included in the studies. Among them, 137 were active athletes in the test group, while 116 were sedentary or inactive individuals in the control group.

All studies analyzed saliva samples. Specifically, two studies examined stimulated saliva using a paraffin block, one study analyzed unstimulated saliva, and two studies did not specify the type of saliva analyzed. All studies focused on bacterial detection within the salivary microbiota. Among the methodologies employed, one study used polymerase chain reaction (PCR) [21] for microbiota detection, two studies used agar culture methods [18, 19], and three studies used 16s rRNA sequencing techniques [17,19,20].

The criteria for dental assessments included the Decayed, Missing, and Filled Teeth (DMF) Index [17, 19, 21]. Significant Caries Index (SiC) [17], Bleeding on probing (BOP) [17], Plaque Index (Pi) [17,19], Loe-Silness Index (LSI) [19], Loe Silness Gingival Index [19], as well as evaluations for dental stains, erosions, trauma, aphthae, anomalies [21], fluoride intake [21], and Saliva pH [19]. Notably, no dental check-up was conducted in the study of Lamb et al. [18] and Kalabiska et al. [20].

To reduce potential biases, the studies collected data on dietary habits and supplementation [19, 21], general oral quality of life [19], dental visit history [19], oral hygiene practices and health behaviors [19, 21], and stress [18,19]. Additionally, height, body weight [17-19], and body mass index (BMI) were analyzed [18,19].

According to fitness classification, only one study assessed maximal oxygen consumption (VO_2_ max) to discriminate between in-season and off-season participants. Furthermore, two studies investigated salivary IgA levels [18, 21], and one study investigated cortisol levels [18] in addition to oral microbiota detection.

Bias and Quality of the Included Studies

Based on the criteria outlined in the NIH study quality assessment tools for case-control and cross-sectional studies, three out of the five studies were qualified as having a low risk of bias [18,19,21], while two studies [17,20] were classified as having a medium risk of bias. The quality assessment results for each selected study are detailed in Table 3 and Table 4.

**Table 3:** The NIH quality assessment tool for case-control studies NIH: National Institutes of Health; Y: yes; N: No; NA: not applicable

NIH quality assesment for case-control studies	Research question	Study population	Sample size justification	Groups recruited from the same population	Inclusion and exclusion criteria respecified and applied uniformly	Case and control definitions	Random selection of study participants	Concurrent controls	Exposure assessed prior to outcome measurement	Exposure measures and assessment	Blinding of exposure assessors	Statistical analysis
	1	2	3	4	5	6	7	8	9	10	11	12
Lamb et al., 2016 [18]	Y	Y	N	Y	Y	Y	NA	Y	Y	N	N	N
Minty et al., 2018 [19]	Y	Y	N	Y	Y	Y	NA	Y	Y	N	N	Y
Kalabiska et al., 2023 [20]	Y	Y	N	Y	Y	N	NA	Y	Y	N	N	N
D’Ercole et al., 2015 [21]	Y	Y	N	Y	Y	Y	NA	Y	Y	Y	N	Y

**Table 4:** The NIH quality assessment tool for cross-sectional studies NIH: National Institutes of Health; Y: yes; N: No; NA: not applicable

NIH quality assesment for cross-sectional studies	Research question	Study population	Study population	Groups recruited from the same population	Sample size justification	Exposure assessed prior to outcome measurement	Sufficient timeframe to see an effect	Different levels of exposure of interest	Exposure measures and assessment	Repeated exposure assessment	Outcome measures	Blinding of outcome assessors	Follow-up rate	Statistical analysis
	1	2	3	4	5	6	7	8	9	10	11	12	13	14
Basak et al., 2015 [17]	Y	Y	NA	Y	N	N	N	N	Y	N	Y	NR	NA	N

The Oral Microbiota in Relation to Aquatic Sports Setting

The key results obtained from the studies are presented in Table 5. 

**Table 5:** Principal outcomes of the selected studies

Study name	Main results
Basak et al., 2015 [17]	The study identified *Streptococcus, Pseudomonas,* and other genera at relatively high levels in competitive swimmers. Post training, *Stenotrophomonas *increased in some, but not in all athletes.
Lamb et al., 2016 [18]	Levels of *Streptococcus*, particularly *Streptococcus mitis *and *Streptococcus salivarius*, were found to be lower in the group of active female hockey players.
Minty et al., 2018 [19]	Rugby players exhibited increased levels of *Streptococcus *species, including *Streptococcus mutans, Streptococcus thermophilus, Streptococcus sobrinus*, and *Streptococcus gordonii.* Additionally, the abundance of *Rothia *was higher in rugby players. No significant differences were observed in *Staphylococcus *levels.
Kalabiska et al., 2023 [20]	Water polo players showed increased *Veillonella* and decreased *Gemella *compared to non-athletes.
D’Ercol et al., 2015 [21]	Non-competitive swimmers had a significantly higher prevalence of *Streptococcus sobrinus *and *Streptococcus mutans* was similar in both groups, while *Streptococcus sanguinis* was only found in competitive swimmers.

In the three studies examining water sports, salivary samples were used to analyze the oral microbiota. Basak et al. [17] and Kalabiska et al. [20] utilized sequencing techniques, while D'Ercole et al. used PCR methods [21].

The findings across these studies were heterogeneous, particularly regarding bacterial genus. In the study by Basak et al. on competitive swimmers, the genera *Streptococcus *(p=0.232), *Pseudomonas *(p=0.526), *Serratia *(p=1.000), *Chryseobacterium *(p=0.372), and *Rothia *(p=0.052) were observed at relatively high levels compared to other genera [17]. No significant variations were observed post-training, except for *Stenotrophomonas *(p=0.013), which showed a notable increase, though this was not consistent across all athletes and was reduced in some participants.

Kalabiska et al. revealed an increase in the *Veillonella *genus and a decrease in the *Gemella *genus in water polo players compared with sedentary players (p < 0.05) [20].

The study by D'Ercole et al., focusing on competitive and non-competitive swimmers, specifically examined the *Streptococcus *genus and the species *Streptococcus mutans*, *Streptococcus sobrinus*, and *Streptococcus sanguinis* [21]. They found that *Streptococcus mutans* was present in 18.64% of competitive swimmers and 32.2% of non-competitive swimmers, although this difference was not significant (p > 0.05). The only significant finding was for *Streptococcus sobrinus*, which appeared in 22.03% of competitive swimmers and 91.6% of non-competitive swimmers (p < 0.05); both *Streptococcus mutans* and *Streptococcus sobrinus* were linked to dental caries development. Additionally, *Streptococcus sanguinis*, a bacteria associated with dental health, was exclusively detected in competitive swimmers. Basak et al. [17] noted variations at the species level post training, but none were statistically significant.

Only Basak et al. assessed bacterial diversity, finding no significant differences before and after training for the Shannon Index (p = 0.54), Simpson Index (p = 0.784), and the Inverse Simpson Index (p = 0.747) [17]. 

The Oral Microbiota in Relation to Team Sport Setting

In the two studies focusing on team sports, salivary samples were used to analyze the oral microbiota. Both Lamb et al. [18] and Minty et al. [19] used cultured bacteria on agar. Minty et al. also performed a sequencing method to further characterize the salivary microbiota. Notable variations in oral microbiota composition were observed between female hockey players and professional rugby players [19].

Lamb et al. [18] reported an increase in the overall bacterial count in female hockey players, while Minty et al. observed a decrease in this parameter among professional rugby players [19]. Specifically, Lamb et al. found a reduction in the *Streptococcus *genus, particularly in *Streptococcus *​​​​​*mitis *and *Streptococcus* ​​​​​​*salivarius*, in female hockey players compared to their off-season counterparts [18]. In contrast, Minty et al. identified an increase in *Streptococcus *in rugby players, notably *Streptococcus mutans*, *Streptococcus *​​​​​*thermophilus*, *Streptococcus *​​​​​*sobrinus*, and *Streptococcus gordonii*, all of which are associated with dental caries [19]. Additionally, rugby players exhibited a higher abundance of *Rothia *genus compared with sedentary players. No differences were observed between the two groups in the presence of *Staphylococcus*.

Finally, the overall bacterial diversity was found to be lower in professional rugby players than in non-professional athletes [19].

Discussion

Understanding the Modifications in the Oral Microbiota Associated With Sports Practice

This review synthesizes recent findings on the association between oral microbiota composition and physical activity. Current evidence suggests that individuals engaged in regular physical activity demonstrate distinct oral microbiota composition profiles compared to sedentary or non-active controls. The genus *Streptococcus *is frequently modified in athletes [18-21] (Table 2). *Streptococcus *species play a major role in the early stages of dental plaque formation, comprising about 60% to 90% of initial supragingival biofilm within the first 24 hours of colonization [22]. *Streptococcus mitis*, *Streptococcus gordoni*, *Streptococcus sanguinis* are primary colonizers on tooth surfaces, whereas *Streptococcus thermophilus*, a lactic-acid-producing bacterium, can thus participate in the development of caries by the acidification of the environment [23,24]. Cariogenic species, such as *Streptococcus mutans* and *Streptococcus sobrinus*, play central roles in dental caries pathogenesis, while *Streptococcus salivarius* exhibits the potential to inhibit *Streptococcus mutans* biofilm growth [25].

D’Ercole et al.'s study [21] suggests that sports practice may confer beneficial effects on the oral microbiota composition by reducing bacteria linked to periodontal diseases and caries, such as *Streptococcus sobrinus*, *Streptococcus mitis*, and *Streptococcus salivarius*, and by increasing caries-protective species like *Streptococcus sanguinis*. In contrast, other studies indicate that athletic activity could promote the proliferation of cariogenic bacteria, including *Streptococcus mutans*, *Streptococcus sobrinus*, *Streptococcus gordonii*, and *Streptococcus thermophilus* [19,20], especially athletes with a high-carbohydrate diet and insufficient oral hygiene practices may be particularly susceptible. The oral microbiome of athletes revealed a significant increase in the genera *Rothia *[19], *Stenotrophomonas *[17], *Veillonella *[20], and a decrease in the genus *Gemella *[20]. 

In addition, dietary nitrates (NO3-), when ingested, are partly redistributed to saliva and serve as substrates for oral microbial metabolism. This metabolic process reduces NO3- to nitrites (NO2-), involving bacteria capable of nitrate reduction, such as *Veillonella*, *Actinomyces*, *Rothia*, *Neisseria*, *Haemophilus*, and *Streptococcus *[26]. Burleigh et al. found that the rate of nitrate-to-nitrite reduction correlates with the abundance of nitrate-reducing oral bacteria via the nitrate reductase enzyme [27]. Other studies indicate that physical training, such as swimming, can elevate nitrate-reducing bacteria, including *Veillonella*, *Rothia*, and *Staphylococcus*, within the oral microbiota of athletes [28]. These findings suggest that the oral bacteria may play a significant role in catalyzing nitrate reduction, possibly contributing to an increase in nitrite levels following training.

While studies predominantly focus on the impact of exercise on the gut microbiota, the established bidirectional interaction between the oral and gut microbiota suggests parallel influences of physical activity on both microbial communities. For instance, Sabater et al. found a slight increase in *Veillonella *species, including *Veillonella atypica* in the gut microbiota of endurance athletes (marathon runners and rowers), which may be associated with reduced muscular fatigue [29]. Additionally, recent evidence indicates a rise in short-chain fatty acid (SCFA)-producing bacteria, such as *Akkermansia*, *Faecalibacterium*, *Veillonella*, and *Roseburia*, among physically active individuals and those undergoing exercise interventions [30].

The heterogeneity of study methodologies and participant characteristics poses challenges for direct comparisons. For instance, the physical demands and training hours differ between professional rugby players [19] and amateur female hockey players [18]. Future research could benefit from the establishment of objective fitness indicators (e.g., VO_2_ max) to enhance study comparability. Evidence suggests that moderate physical activity promotes protective microbial diversity and oral health, whereas intense training may induce physiological stress that could lead to the alteration of the oral microbiota composition. Athletes, usually considered healthier than the normal population, are in fact more prone to infections of the respiratory tract in the time frames subsequent to heavy sports sessions [31]. This period of vulnerability has been theorized as the ‘open window’ theory characterized by a short-term decreased immune function following endurance training [32,33]. 

This immune alteration might contribute to oral dysbiosis. Additional factors potentially impacting the oral microbiota include dietary changes, shifts in oral pH due to increased respiration, and dehydration associated with intense training [34].

While current findings indicate the oral microbiota of athletes is distinct, more research is needed to elucidate the specific impacts of physical activity. Moreover, many confounding factors, such as diet and hydration status, which are known to influence athletes’ oral health, remain underexplored in existing studies.

Limitations of the present studies

The studies reviewed have several limitations that affect the reliability and generalizability of their findings. One major constraint lies in the small sample sizes across the studies, which limits their statistical power and increases the possibility of false associations. For example, Lamb et al. [18] studied only 10 athletes with nine non-athletes, while Basak et al. [17] analyzed the microbiota in 20 Turkish professional swimmers, and Minty et al. [19] included 24 athletes with 22 non-athletes. Kalabiska et al. [20] had a larger sample size, with 124 participants, but for financial reasons, only 29 athletes and 16 non-athletes were analyzed for salivary microbiological composition. Only the study by D’Ercole et al. involved a sample size sufficient to draw stronger conclusions, with 123 participants, including 54 athletes and 69 non-athletes [20]. Despite this, the limited cohorts across studies reduce the scope of conclusions and limit the generalizability of results to broader populations.

In addition to sample size, the diversity of participant characteristics introduces complexity. Significant variability exists across studies regarding gender and age. For example, Lamb et al. [18] recruited only female athletes who participated in different sports (hockey vs. football) in different seasons of the year (in-season vs. off-season). Whereas Minty et al. [19] focused solely on male rugby players, making gender a confounding factor that complicates cross-study comparisons. The oral microbiota differs greatly between men and women, making it impossible to compare studies with exclusively male and female cohorts [35]. This variability is further compounded by the considerable range of ages among participants, from six to 32 years old, from children and adolescents to young adults, as seen in D’Ercole et al. [21] (ages six to 15), Lamb et al. [18] (ages 18 to 20), Kalabiska et al. [20] (ages 16 to 20), and Minty et al. [19] (ages 15 to 32). The oral microbiota undergoes continuous changes during childhood and adolescence due to various physiological factors (permanent teeth, puberty, hormonal changes, etc), making it challenging to directly compare the microbiota of children, adolescents, and adults [36].

Moreover, differences in the sports studied further complicate comparisons. The reviewed studies investigated team sports, such as rugby, hockey, and football, as well as individual water sports like swimming and water polo. Each sport’s physical and environmental demands can significantly affect the oral microbiota composition. For instance, the physiological and energy demands of a professional rugby player may differ greatly from those of a student hockey player or swimmer. Furthermore, water sports, where athletes train extensively in chlorinated environments (e.g., water polo, swimmers). Consequently, the differences in sports types make it difficult to isolate sport-specific impacts on oral microbiota across studies.

Another significant limitation is the inconsistent consideration of confounding factors such as diet, stress, and lifestyle, which are known to influence oral health and oral microbiota composition. Only some studies, such as those by Minty et al. [19] and D’Ercole et al. [21], analyzed the diet of athletes using a questionnaire (quantity and frequency of food intake and the type of food), while others, like Lamb et al. [18], Basak et al., and Kalabiska et al. [20], did not include dietary assessment, which introduces potential confounding variables into the analysis. Similarly, Lamb et al. [18] and Minty et al. [19] assessed the stress levels of athletes, which can impact oral health, through the use of a stress score on a 10-point scale or the Generalized Anxiety Disorder scale (GAD-7), other studies omitted this factor. Such variability in controlling for confounding factors complicates efforts to accurately attribute observed microbiota differences to athletic activity alone.

The reliability of the studies’ methods also varies. Only Minty et al. [19] specified that investigators were calibrated and qualified dentists, adding rigor to the dental assessments. However, even in this study, measurement bias may have been introduced since the investigators were unable to be blinded to participants’ athletic identities due to the physical appearance of rugby players during the oral and dental check-ups. In other studies, such as those by Basak et al. [17], Kalabiska et al. [20], D’Ercole et al. [21], and Lamb et al. [18], it remains unclear whether investigators were calibrated, potentially reducing reliability and increasing the risk of observer bias in oral microbiota assessments.

Finally, the differences in microbiota analysis methods across studies limit the ability to compare findings. While Lamb et al. [18] relied solely on agar culture, Minty et al. [19] combined culture with sequencing, and others, such as Basak et al. [17] and Kalabiska et al. [20], used sequencing alone. D'Ercole et al. utilized PCR [21]. Sequencing offers a more comprehensive and accurate view of bacterial diversity compared to the agar culture and PCR. Furthermore, all studies focused exclusively on bacterial populations, obscuring other oral microbiota components, such as fungi and viruses, which also play roles in maintaining oral health and disease dynamics. Saliva was the biological sample analyzed in all studies. However, variations in the sample collection methods were observed, with some studies using stimulated saliva [19,21] and others using unstimulated saliva [17,18,20].

Future research should prioritize larger, more diverse sample sizes and employ more consistent methodologies for the assessment of sports parameters and the application of microbiological techniques across studies. Comprehensive and standardized microbiota analyses that expand beyond bacteria would also provide a fuller understanding of how sports impact oral health.

## Conclusions

In conclusion, although the available evidence is limited, current literature suggests an association between physical activity and alterations in the oral microbiota composition. However, it remains uncertain whether these modifications are beneficial or detrimental to oral health, as no clinical evidence yet confirms a clear direction of these changes. The considerable heterogeneity across studies stemming from differences in methods, participant groups, and covariate considerations makes direct comparisons challenging. Future studies would benefit from standardized methods and more homogeneous participant groups to improve comparability.

Significant biases also persist, including variability in sample sizes, inconsistencies in covariate selection, and a lack of investigator calibration. The predominance of case-control studies limits the strength of the evidence, as this design would benefit from well-designed cohort studies or randomized controlled trials, which would provide more robust data on the influence of physical activity on oral microbiota. By addressing these methodological issues, future research could clarify whether a direct and reliable link exists between the practice of sport and modifications in the oral microbiota, ultimately leading to better-informed recommendations for athletes’ oral health management.
